# 2-Amino­benzimidazolium hydrogen sulfate

**DOI:** 10.1107/S1600536808041706

**Published:** 2008-12-13

**Authors:** Wei You, Ying Fan, Hui-Fen Qian, Cheng Yao, Wei Huang

**Affiliations:** a College of Sciences, Nanjing University of Technology, Nanjing, 210009, People’s Republic of China; b State Key Laboratory of Coordination Chemistry, Nanjing National Laboratory of Microstructures, School of Chemistry and Chemical Engineering, Nanjing University, Nanjing, 210093, People’s Republic of China

## Abstract

In the title salt, C_7_H_8_N_3_^+^·HSO_4_^−^, the benzimdazole ring system is planar [mean deviation 0.0086 (1) Å]. In the crystal, N—H⋯O and O—H⋯O hydrogen-bond inter­actions give rise to a layer motif.

## Related literature

For related compounds, see: El-Medania *et al.* (2003 ▶); Yeşilel *et al.* (2008 ▶).
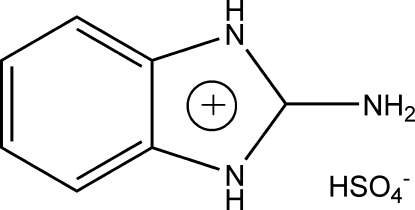


## Experimental

### 

#### Crystal data

C_7_H_8_N_3_^+^·HSO_4_^−^
*M* *_r_* = 231.23
Monoclinic, 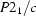

*a* = 10.855 (6) Å
*b* = 13.049 (7) Å
*c* = 7.082 (4) Å
β = 99.025 (7)°
*V* = 990.7 (9) Å^3^
*Z* = 4
Mo *K*α radiation
μ = 0.33 mm^−1^
*T* = 291 (2) K
0.16 × 0.12 × 0.10 mm

#### Data collection

Bruker SMART diffractometer
Absorption correction: multi-scan (*SADABS*; Bruker, 2000 ▶) *T*_min_ = 0.950, *T*_max_ = 0.968
5083 measured reflections
1841 independent reflections
1408 reflections with *I* > 2σ(*I*)
*R*_int_ = 0.057

#### Refinement

*R*[*F* ^2^ > 2σ(*F* ^2^)] = 0.038
*wR*(*F* ^2^) = 0.103
*S* = 0.96
1841 reflections
136 parameters
H-atom parameters constrained
Δρ_max_ = 0.23 e Å^−3^
Δρ_min_ = −0.35 e Å^−3^

### 

Data collection: *SMART* (Bruker, 2000 ▶); cell refinement: *SAINT* (Bruker, 2000 ▶); data reduction: *SAINT*; program(s) used to solve structure: *SHELXS97* (Sheldrick, 2008 ▶); program(s) used to refine structure: *SHELXL97* (Sheldrick, 2008 ▶); molecular graphics: *SHELXTL* (Sheldrick, 2008 ▶); software used to prepare material for publication: *SHELXTL*.

## Supplementary Material

Crystal structure: contains datablocks global, I. DOI: 10.1107/S1600536808041706/ng2522sup1.cif

Structure factors: contains datablocks I. DOI: 10.1107/S1600536808041706/ng2522Isup2.hkl

Additional supplementary materials:  crystallographic information; 3D view; checkCIF report

## Figures and Tables

**Table 1:** Hydrogen-bond geometry (Å, °)

*D*—H⋯*A*	*D*—H	H⋯*A*	*D*⋯*A*	*D*—H⋯*A*
N1—H1*A*⋯O4^i^	0.86	2.00	2.848 (3)	167
O1—H1*B*⋯O2^ii^	0.82	1.80	2.619 (2)	176
N2—H2*A*⋯O3	0.86	1.94	2.795 (2)	177
N3—H3*A*⋯O3^i^	0.86	2.09	2.899 (3)	157
N3—H3*B*⋯O2	0.86	2.17	2.987 (2)	158

Symmetry codes: (i) 
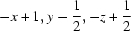
; (ii) 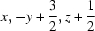.

**Fig. 1.**
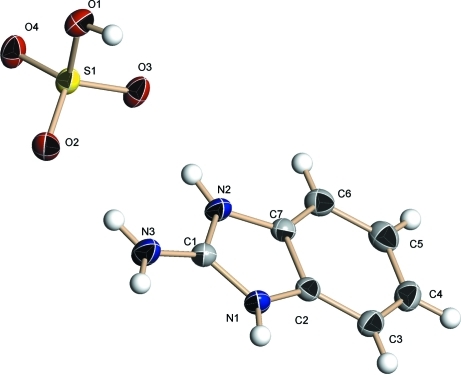
An ORTEP drawing of the title compound with the atom-numbering scheme. Displacement ellipsoids are drawn at the 30% probability level.

**Fig. 2.**
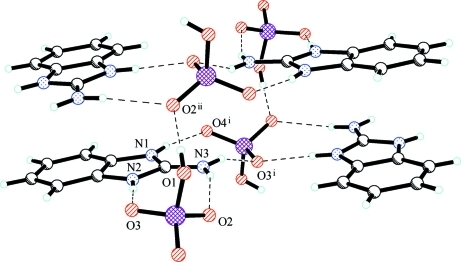
A perspective view of the packing structure of the title compound. Symmetry codes: (i) -x + 1, y - 1/2, -z + 1/2; (ii) x, -y + 3/2, z + 1/2.

## supplementary crystallographic information

### Comment

Several ion-pair adducts of 2-aminobenzimidazole with different organic acids such as picric acid (El-Medania *et al.*, 2003) and squaric acid (YeŞilel *et al.*, 2008) have been reported. Herein, we present a hydrogen sulfate of 2-aminobenzimidazole.

The atom-numbering scheme of the title compound is shown in Fig. 1, while selected bond distances and bond angles are given in Table 1. The benzimidazole skeleton of the title compound is planar and the proton is delocalized within the imidazole ring although it is added to one of the nitrogen atoms. With regard to the hydrogen sulfate anion, the hydrogen atom is added to the O1 atom of SO_4_ group due to the obviously longer O1–S1 bond length. In the crystal packing, typical *π-π* stacking can be found between neighbouring aromatic rings with the centroid-to-centroid separation of 3.452 (2) Å. Furthermore, N—H···O and O—H···O hydrogen bonding interactions are found between adjacent molecules to form a three-dimensional network (Fig. 2).

### Experimental

The treatment of 2-aminobenzimidazole dissolved in methanol with an excess of hydrochloric acid yields the title compound. Single crystal suitable for X-ray diffraction measurement was obtained after 3 days' slow evaporation of the mother liquid at room temperature in air. Anal. Calcd. For C~7~H~9Ñ~3Õ~4~S: C, 36.36; H, 3.92; O, 27.68%. Found: C, 36.17; H, 4.03; N, 27.74%. Main FT—IR absorptions (KBr pellets, cm^-1^): 3385 (*s*), 3194 (*m*), 1687 (*s*), 1476 (*m*), 1286 (*m*), 1206(*s*), 1175 (*vs*), 1070 (*m*), 1026 (*m*), 888 (*s*), and 577 (*w*).

### Refinement

The non-hydrogen atoms were refined anisotropically, whereas the H atoms bonded with carbon, nitrogen and oxygen atoms were placed in geometrically idealized positions (C—H = 0.93 Å, N—H = 0.86 Å and O—H = 0.82 Å) and refined as riding atoms, with *U*_iso_(H) = 1.2*U*_eq_(C) and 1.2*U*~eq~(N) and *U*ĩso~(H) = 1.5*U*~eq~(O).

### Crystal data

**Table:**

C_7_H_8_N_3_^+^·HSO_4_^−^	*F*(000) = 480
*M**_r_* = 231.23	*D*_x_ = 1.550 Mg m^−^^3^
Monoclinic, *P*2_1_/*c*	Mo *K*α radiation, λ = 0.71073 Å
Hall symbol: -P 2ybc	Cell parameters from 1841 reflections
*a* = 10.855 (6) Å	θ = 1.0–1.0°
*b* = 13.049 (7) Å	µ = 0.33 mm^−^^1^
*c* = 7.082 (4) Å	*T* = 291 K
β = 99.025 (7)°	Block, colourless
*V* = 990.7 (9) Å^3^	0.16 × 0.12 × 0.10 mm
*Z* = 4	

### Data collection

**Table:**

Bruker SMART diffractometer	1841 independent reflections
Radiation source: fine-focus sealed tube	1408 reflections with *I* > 2σ(*I*)
graphite	*R*_int_ = 0.057
φ and ω scans	θ_max_ = 25.5°, θ_min_ = 2.5°
Absorption correction: multi-scan (*SADABS*; Bruker, 2000)	*h* = −13→13
*T*_min_ = 0.950, *T*_max_ = 0.968	*k* = −11→15
5083 measured reflections	*l* = −8→8

### Refinement

**Table:**

Refinement on *F*^2^	Secondary atom site location: difference Fourier map
Least-squares matrix: full	Hydrogen site location: inferred from neighbouring sites
*R*[*F*^2^ > 2σ(*F*^2^)] = 0.038	H-atom parameters constrained
*wR*(*F*^2^) = 0.103	*w* = 1/[σ^2^(*F*_o_^2^) + (0.0593*P*)^2^] where *P* = (*F*_o_^2^ + 2*F*_c_^2^)/3
*S* = 0.96	(Δ/σ)_max_ = 0.001
1841 reflections	Δρ_max_ = 0.23 e Å^−^^3^
136 parameters	Δρ_min_ = −0.35 e Å^−^^3^
0 restraints	Extinction correction: *SHELXL97* (Sheldrick, 2008)
Primary atom site location: structure-invariant direct methods	Extinction coefficient: 0

### Special details

**Table:**

Experimental. The structure was solved by direct methods (Bruker, 2000) and successive difference Fourier syntheses.
Geometry. All e.s.d.'s (except the e.s.d. in the dihedral angle between two l.s. planes) are estimated using the full covariance matrix. The cell e.s.d.'s are taken into account individually in the estimation of e.s.d.'s in distances, angles and torsion angles; correlations between e.s.d.'s in cell parameters are only used when they are defined by crystal symmetry. An approximate (isotropic) treatment of cell e.s.d.'s is used for estimating e.s.d.'s involving l.s. planes.
Refinement. Refinement of *F*^2^ against ALL reflections. The weighted *R*-factor *wR* and goodness of fit *S* are based on *F*^2^, conventional *R*-factors *R* are based on *F*, with *F* set to zero for negative *F*^2^. The threshold expression of *F*^2^ > σ(*F*^2^) is used only for calculating *R*-factors(gt) *etc*. and is not relevant to the choice of reflections for refinement. *R*-factors based on *F*^2^ are statistically about twice as large as those based on *F*, and *R*- factors based on ALL data will be even larger.

### Fractional atomic coordinates and isotropic or equivalent isotropic displacement parameters (Å^2^)

**Table:**

	*x*	*y*	*z*	*U*_iso_*/*U*_eq_	
C1	0.61393 (19)	0.67017 (16)	0.3926 (3)	0.0400 (5)	
C2	0.80252 (18)	0.71967 (16)	0.5357 (3)	0.0387 (5)	
C3	0.9247 (2)	0.72581 (18)	0.6229 (3)	0.0483 (6)	
H3	0.9725	0.6674	0.6557	0.058*	
C4	0.9730 (2)	0.82273 (19)	0.6594 (3)	0.0533 (6)	
H4	1.0555	0.8296	0.7178	0.064*	
C5	0.9026 (2)	0.91056 (19)	0.6120 (3)	0.0563 (6)	
H5	0.9382	0.9746	0.6407	0.068*	
C6	0.7787 (2)	0.90386 (17)	0.5216 (3)	0.0497 (6)	
H6	0.7310	0.9620	0.4866	0.060*	
C7	0.73133 (18)	0.80702 (16)	0.4872 (3)	0.0385 (5)	
N1	0.72523 (15)	0.63593 (13)	0.4769 (2)	0.0410 (4)	
H1A	0.7460	0.5725	0.4925	0.049*	
N2	0.61415 (15)	0.77282 (13)	0.3989 (2)	0.0429 (5)	
H2A	0.5521	0.8116	0.3555	0.051*	
N3	0.51968 (16)	0.61327 (14)	0.3154 (3)	0.0534 (5)	
H3A	0.5265	0.5476	0.3163	0.064*	
H3B	0.4511	0.6416	0.2639	0.064*	
O1	0.21109 (12)	0.84704 (11)	0.3128 (2)	0.0508 (4)	
H1B	0.2450	0.8155	0.4069	0.076*	
O2	0.32882 (14)	0.75437 (11)	0.1058 (2)	0.0533 (5)	
O3	0.41587 (12)	0.90419 (10)	0.2672 (2)	0.0494 (4)	
O4	0.23730 (14)	0.91989 (11)	0.0168 (2)	0.0539 (4)	
S1	0.30277 (5)	0.85757 (4)	0.16657 (8)	0.0404 (2)	

### Atomic displacement parameters (Å^2^)

**Table:**

	*U*^11^	*U*^22^	*U*^33^	*U*^12^	*U*^13^	*U*^23^
C1	0.0399 (11)	0.0385 (12)	0.0414 (12)	0.0044 (9)	0.0057 (10)	−0.0029 (9)
C2	0.0419 (12)	0.0400 (12)	0.0344 (11)	0.0037 (9)	0.0069 (9)	−0.0013 (9)
C3	0.0444 (12)	0.0581 (15)	0.0420 (13)	0.0089 (11)	0.0057 (10)	0.0033 (11)
C4	0.0416 (13)	0.0658 (17)	0.0518 (15)	−0.0066 (11)	0.0051 (11)	−0.0039 (12)
C5	0.0584 (15)	0.0548 (16)	0.0577 (15)	−0.0130 (12)	0.0151 (12)	−0.0092 (12)
C6	0.0537 (14)	0.0407 (13)	0.0552 (15)	0.0009 (10)	0.0103 (12)	−0.0020 (11)
C7	0.0401 (11)	0.0386 (12)	0.0371 (12)	0.0045 (9)	0.0073 (9)	−0.0016 (9)
N1	0.0438 (10)	0.0322 (10)	0.0453 (11)	0.0091 (7)	0.0017 (8)	−0.0001 (7)
N2	0.0404 (10)	0.0343 (10)	0.0520 (12)	0.0109 (7)	0.0013 (8)	−0.0010 (8)
N3	0.0447 (10)	0.0400 (11)	0.0719 (14)	0.0050 (8)	−0.0019 (10)	−0.0067 (10)
O1	0.0408 (8)	0.0495 (10)	0.0611 (10)	0.0055 (7)	0.0047 (8)	0.0063 (7)
O2	0.0640 (10)	0.0331 (9)	0.0582 (10)	0.0091 (7)	−0.0047 (8)	−0.0073 (7)
O3	0.0387 (8)	0.0372 (9)	0.0669 (10)	−0.0035 (6)	−0.0086 (7)	0.0032 (7)
O4	0.0609 (9)	0.0381 (9)	0.0548 (10)	0.0046 (7)	−0.0153 (8)	0.0072 (7)
S1	0.0402 (3)	0.0288 (3)	0.0484 (4)	0.0019 (2)	−0.0045 (2)	0.0007 (2)

### Geometric parameters (Å, °)

**Table:**

C1—N3	1.312 (3)	C6—C7	1.372 (3)
C1—N1	1.338 (2)	C6—H6	0.9300
C1—N2	1.340 (3)	C7—N2	1.400 (2)
C2—C3	1.375 (3)	N1—H1A	0.8600
C2—C7	1.390 (3)	N2—H2A	0.8600
C2—N1	1.401 (3)	N3—H3A	0.8600
C3—C4	1.378 (3)	N3—H3B	0.8600
C3—H3	0.9300	O1—S1	1.5510 (18)
C4—C5	1.389 (3)	O1—H1B	0.8200
C4—H4	0.9300	O2—S1	1.4549 (16)
C5—C6	1.398 (3)	O3—S1	1.4528 (14)
C5—H5	0.9300	O4—S1	1.4336 (15)
			
N3—C1—N1	126.0 (2)	C6—C7—N2	131.49 (19)
N3—C1—N2	125.21 (19)	C2—C7—N2	106.26 (18)
N1—C1—N2	108.79 (18)	C1—N1—C2	109.21 (17)
C3—C2—C7	121.5 (2)	C1—N1—H1A	125.4
C3—C2—N1	132.04 (19)	C2—N1—H1A	125.4
C7—C2—N1	106.42 (17)	C1—N2—C7	109.28 (16)
C2—C3—C4	116.7 (2)	C1—N2—H2A	125.4
C2—C3—H3	121.7	C7—N2—H2A	125.4
C4—C3—H3	121.7	C1—N3—H3A	120.0
C3—C4—C5	122.2 (2)	C1—N3—H3B	120.0
C3—C4—H4	118.9	H3A—N3—H3B	120.0
C5—C4—H4	118.9	S1—O1—H1B	109.5
C4—C5—C6	120.8 (2)	O4—S1—O3	114.09 (9)
C4—C5—H5	119.6	O4—S1—O2	113.76 (10)
C6—C5—H5	119.6	O3—S1—O2	110.13 (9)
C7—C6—C5	116.5 (2)	O4—S1—O1	104.41 (10)
C7—C6—H6	121.8	O3—S1—O1	106.91 (10)
C5—C6—H6	121.8	O2—S1—O1	106.88 (10)
C6—C7—C2	122.2 (2)		

### Hydrogen-bond geometry (Å, °)

**Table:**

*D*—H···*A*	*D*—H	H···*A*	*D*···*A*	*D*—H···*A*
N1—H1A···O4^i^	0.86	2.00	2.848 (3)	167
O1—H1B···O2^ii^	0.82	1.80	2.619 (2)	176
N2—H2A···O3	0.86	1.94	2.795 (2)	177
N3—H3A···O3^i^	0.86	2.09	2.899 (3)	157
N3—H3B···O2	0.86	2.17	2.987 (2)	158

Symmetry codes: (i) −*x*+1, *y*−1/2, −*z*+1/2; (ii) *x*, −*y*+3/2, *z*+1/2.
